# *Ovicides paralithodis* (Nemertea, Carcinonemertidae), a new species of symbiotic egg predator of the red king crab *Paralithodes camtschaticus* (Tilesius, 1815) (Decapoda, Anomura)

**DOI:** 10.3897/zookeys.258.4260

**Published:** 2013-01-14

**Authors:** Hiroshi Kajihara, Armand M. Kuris

**Affiliations:** 1 Faculty of Science, Hokkaido University, Sapporo 060-0810, Japan; 2 Marine Science Institute & Department of Ecology, Evolution and Marine Biology, University of California, Santa Barbara, CA 93106-9610, USA

**Keywords:** Nemertini, Crustacea, *Paralithodes camtschatica*, symbiont, egg predator

## Abstract

*Ovicides paralithodis* **sp. n.** is described from the egg mass of the red king crab *Paralithodes camtschaticus* (Tilesius, 1815) from the Sea of Okhotsk, off Hokkaido, Japan, and Alaska, USA. Among four congeners, *Ovicides paralithodis* can be distinguished from *Ovicides julieae* Shields, 2001 and *Ovicides davidi* Shields and Segonzac, 2007 by having no eyes; from *Ovicides jonesi* Shields and Segonzac, 2007 by the presence of basophilic, vacuolated glandular lobes in the precerebral region; and from *Ovicides jasoni* Shields and Segonzac, 2007 by the arrangement of the acidophilic submuscular glands, which are not arranged in a row. *Ovicides paralithodis* represents the third described species of egg-predatory nemertean from *Paralithodes camtschaticus*, the second described carcinonemertid species from Japan, and the 21st described species in the family. The intensity of infestations may exceed 24,000 worms per a single egg-bearing pleopod of *Paralithodes camtschaticus*. A preliminary molecular phylogenetic analysis based on sequences of 28S rRNA and cytochrome *c* oxidase subunit I genes among selected monostiliferous hoplonemertean species supported the monophyly of Carcinonemertidae, suggesting that within the lineage of the family, evolution of the unique vas deferens, Takakura’s duct, preceded loss of accessory stylets and accessory-stylet pouches.

## Introduction

Nemerteans in the monostiliferous hoplonemertean family Carcinonemertidae are ectosymbiont egg predators of decapod crustacean hosts (Humes 1942, Jensen and Sadeghian 2005). The family is comprised of two genera, *Carcinonemertes* Coe, 1902 and *Ovicides* Shields, 2001, each containing 16 (Sadeghian and Santos 2010) and four (Shields and Segonzac 2007) species, respectively. They are known from approximately 70 host species (Sadeghian and Santos 2010), but the actual diversity of carcinonemertids is likely to be much greater (Kuris 1993). Crustacean-egg predatory nemerteans other than Carcinonemertidae include *Alaxinus oclairi* Gibson, Wickham and Kuris, 1990 and *Pseudocarcinonemertes homari* Fleming and Gibson, 1981.

The red king crab, *Paralithodes camtschaticus* (Tilesius, 1815), is a commercially important anomuran decapod, native to the Bering Sea, the Sea of Japan, the Sea of Okhotsk, and the North Pacific from the Kamchatka Peninsula to Alaska. Wickham and Kuris (1985) listed three undescribed species of egg-predator nemerteans on *Paralithodes camtschaticus* in Alaska, and Wickham and Kuris (1988) recognized five undescribed forms. Later, Forms 1 and 2 *sensu* Wickham and Kuris (1988) were respectively described as *Carcinonemertes regicides* Shields, Wickham and Kuris, 1989 and *Alaxinus oclairi*, while Forms 3–5 remained undescribed.

A survey of egg masses of *Paralithodes camtschaticus* in Hokkaido, northern Japan, yielded specimens that correspond to Form 4 of Wickham and Kuris (1988) from Alaska, which is herein described as a new species belonging to *Ovicides*.

## Methods

Twenty female specimens of the red king crab *Paralithodes camtschaticus* were obtained in the Sea of Okhotsk, off Abashiri, Hokkaido, Japan, at 44°06'N, 144°32'E, from 215 m in depth, by crab cages set from 28 November 2011 to 15 December 2011. Of these female crabs, 16 were ovigerous, from three of which we procured a single nemertean specimen. The worms were anaesthetized in MgCl_2_ solution isotonic to seawater. The anterior halves of the worms were fixed in Bouin’s solution for histological preparation; the posterior halves were preserved in 99% ethanol for DNA extraction. Histological preparation follows that of Kajihara et al. (2011a, b). The type slides are deposited in the Hokkaido University Museum, Sapporo, Japan (ZIHU).

DNA extraction, PCR amplification, and sequencing of the nuclear 28S rRNA gene and mitochondrial cytochrome *c* oxidase subunit I gene (COI) largely follow those of Kajihara et al. (2011a, b). Sequences from the holotype, the egg strand laid by the holotype, and the allotype were exactly the same (*p* = 0.0), with respect to both 28S rRNA (1141 bp) and COI (658 bp).

A preliminary analysis was carried out to assess the phylogenetic affinities of the new species, including 16 species of Distromatonemertea, in addition to two outgroup species, for which 28S rRNA and COI sequences were available in GenBank (Table 1). Alignment of the sequences was carried out by MUSCLE (Edgar 2004a, b) implemented in MEGA ver. 5.05 (Tamura et al. 2011). Model selection and a maximum likelihood analysis using nearest-neighbour interchange tree rearrangement in heuristic search were also performed by MEGA ver. 5.05 (Tamura et al. 2011), based on the general time-reversible model (Tavaré 1986) with gamma-distributed rate heterogeneity and a proportion of invariant sites (GTR + G + I) selected by Akaike Information Criterion (Akaike 1974) as the best-fit substitution model; a bootstrap analysis (Felsenstein 1985) with 1000 replications was performed to evaluate nodal supports. The concatenated matrix of 28S rRNA and COI sequences comprised 1851 bp (excluding gap positions) after alignment of each submatrix.

**Table 1.** List of species included in the phylogenetic analysis, with GenBank accession numbers.<br/>

**Species**	**28S rRNA**	**COI**	**Sources**
*Amphiporus imparispinosus* Griffin, 1898	HQ856878	HQ848612	Andrade et al. (2012)
*Amphiporus lactifloreus* (Johnston, 1828)	HQ856876	HQ848611	Andrade et al. (2012)
*Antarctonemertes varvarae* Chernyshev, 1999	AJ436845	AJ436900	Thollesson and Norenburg (2003)
*Argonemertes australiensis* (Dendy, 1892)	HQ856892	HQ848601	Andrade et al. (2012)
*Carcinonemertes carcinophila* (Kölliker, 1845)	HQ856893	HQ848619	Andrade et al. (2012)
*Carcinonemertes* cf. *carcinophila imminuta* Humes, 1942	AJ436846	AJ436901	Thollesson and Norenburg (2003)
*Emplectonema gracile* (Johnston, 1837)	HQ856883	HQ848620	Andrade et al. (2012)
*Gononemertes parasita* Bergendal, 1900	HQ856889	HQ848607	Andrade et al. (2012)
*Leptonemertes chalicophora* (Graff, 1879)	HQ856898	HQ848596	Andrade et al. (2012)
*Nemertellina yamaokai* Kajihara et al., 2000	AJ436852	AJ436907	Thollesson and Norenburg (2003)
*Oerstedia dorsalis* (Abildgaard, 1806)	AY210465	AY791971	Thollesson and Norenburg (2003)
*Oerstedia venusta* Iwata, 1954	AJ436856	AJ436911	Thollesson and Norenburg (2003)
*Ovicides paralithodis* sp. n.	AB704416	AB704417	Present study
*Paranemertes peregrina* Coe, 1901	AJ436860	AJ436915	Thollesson and Norenburg (2003)
*Paranemertes sanjuanensis* Stricker, 1982	AJ436862	AJ436917	Thollesson and Norenburg (2003)
*Zygonemertes simoneae* Corrêa, 1961	AJ436867	AJ436922	Thollesson and Norenburg (2003)
*Zygonemertes virescens* (Verrill, 1879)	AJ436868	AJ436923	Thollesson and Norenburg (2003)
Outgroups			
*Nipponnemertes punctatula* (Coe, 1905)	AJ436855	AJ436910	Thollesson and Norenburg (2003)
*Paradrepanophorus crassus* (Quatrefages, 1846)	HQ856867	HQ848603	Andrade et al. (2012)

Observations on abundance and geographic distribution in Alaska were conducted from 1983 to 1985, as described in Kuris et al. (1991). Observations of living specimens were made on worms from red king crabs collected near Homer, Seward and Juneau, Alaska.

## Results

### Ovicides paralithodis sp. n.

urn:lsid:zoobank.org:act:1E52DC7A-C52F-4502-AEAC-7A3EB0244F4D

http://species-id.net/wiki/Ovicides_paralithodis

**Figure 1.**
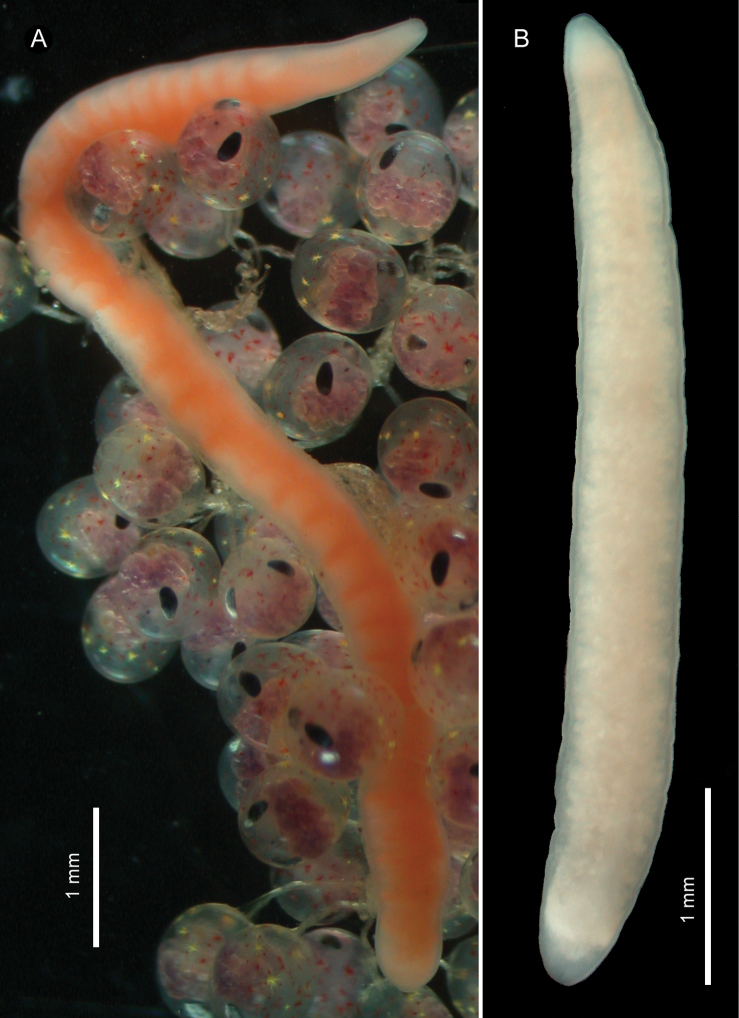
*Ovicides paralithodis* sp. n., photographs taken in life. **A** holotype, female, ZIHU 4271 **B** allotype, male, ZIHU 4272.

Figs 1 [Fig F2] [Fig F3] [Fig F4] –5

Carcinonemertidae Form 4: Wickham and Kuris (1988).

#### Material examined.

Holotype: female, ZIHU 4271, serial transverse sections (8 µm thick) of anterior body fragment, stained with Mallory’s trichrome method, 5 slides. Allotype: male, ZIHU 4272, serial transverse sections (8 µm thick) of anterior body fragment, stained with Mallory’s trichrome method, 3 slides. The other specimen obtained (female) was destroyed and lost during preparation.

#### Diagnosis.

An *Ovicides* without eyes; vacuolated, basophilic glandular lobes extending pre- and post-cerebrally; acidophilic submuscular glands scattered among basophilic lobes, not arranged in row; sexes separate; female and male about 1 cm and 5 mm in length, respectively.

#### Type host.

*Paralithodes camtschaticus* (Tilesius, 1815) (Decapoda, Anomura).

#### Description.

*External features*. In life, holotype (female) about 1 cm long, 0.9 mm wide; pale orange in colour (largely due to alimentary canal), except whitish tip of head (Fig. 1A). Allotype (male) about 5 mm in length, 0.3 mm in width; cream white in colour (Fig. 1B). Living in thin, transparent mucous tube.

**Figure 2. F2:**
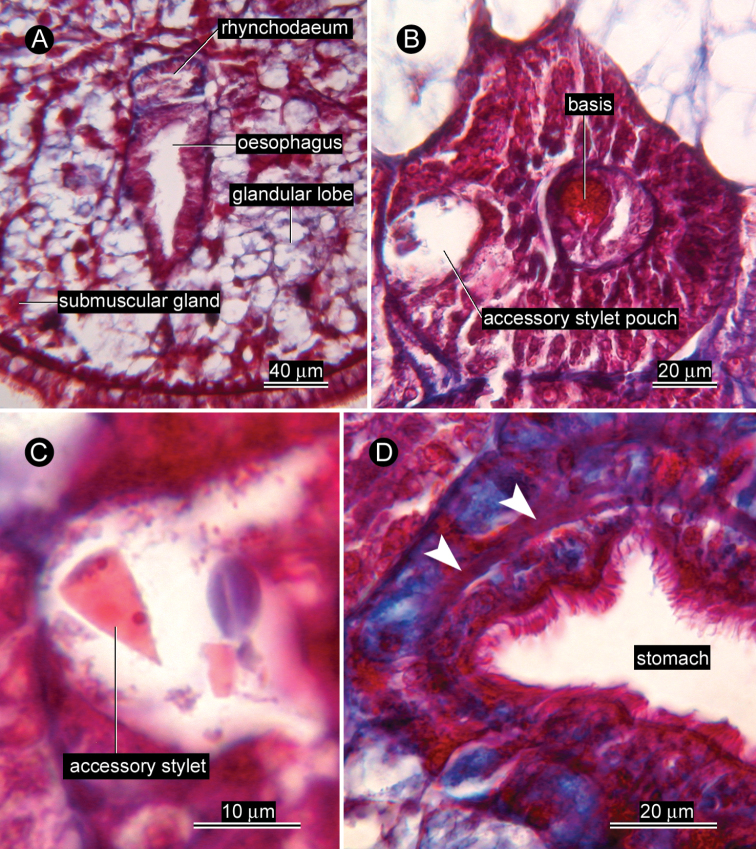
*Ovicides paralithodis* sp. n., photomicrographs of transverse sections. **A** precerebral region, showing rhynchodaeum just after branched off from oesophagus **B** anterior proboscis chamber showing stylet basis and one of the two accessory stylet pouches **C** accessory stylet **D** stomach, showing circular muscle fibres (indicated by arrowheads). A, C, D, allotype, male, ZIHU 4272; B, holotype, female, ZIHU 4271.

**Figure 3. F3:**
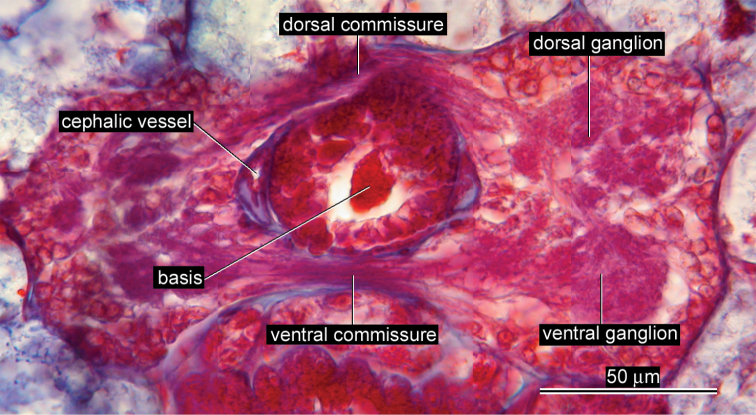
*Ovicides paralithodis* sp. n., photomicrograph of transverse section through brain ring, allotype, male, ZIHU 4272.

*Proboscis apparatus*. Rhynchodaeum opening to dorsal wall of oesophagus (Fig. 2A). Anterior proboscis chamber 136 µm (unknown in allotype) long by 100 µm (82 µm in allotype) diameter; central stylet basis 48 µm (56 µm in allotype) long by 20 µm (20 µm in allotype) diameter (Figs 2B, 3); central stylet 16 µm (12 µm in allotype) in length (all measured from transverse sections); stylet to basis ratio 0.21–0.33; two accessory stylet pouches each containing two accessory stylets (Fig. 2C). Middle proboscis chamber 80 µm (54 µm in allotype) in diameter. Posterior proboscis chamber 240 µm (unknown in allotype) long by 130 µm (94 µm in allotype) wide. Proboscis almost same length as rhynchocoel, extending posteriorly behind pylorus-intestine junction; musculature of rhynchocoel wall uncertain in light microscopy.

*Alimentary canal*. Oesophagus opening ventrally at tip of head. Stomach wall containing circular muscle fibres (Fig. 2D).

**Figure 4. F4:**
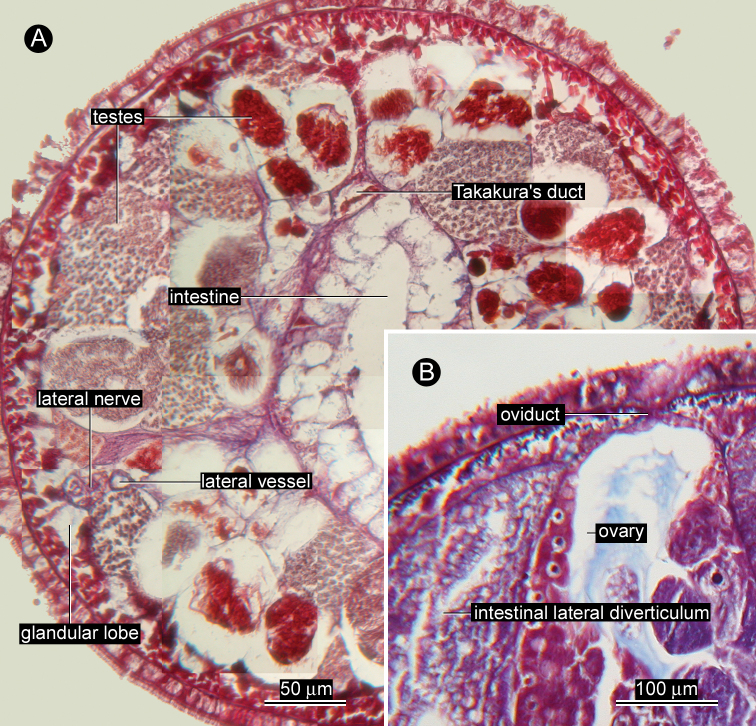
*Ovicides paralithodis* sp. n., photomicrographs of transverse sections through intestinal region. **A** testes and Takakura’s duct, allotype, male, ZIHU 4272 **B** gonopore opening dorsally, holotype, female, ZIHU 4271.

*Glandular system*. Vacuolated, basophilic glandular lobes filling much space of precerebral region between body-wall musculature and oesophagus (Fig. 2A), extending post-cerebrally in intestinal region, but gradually less distinct posteriorly (Fig. 4A). Acidophilic submuscular glands scattered among basophilic lobes (Fig. 2A), not arranged in row beneath body-wall musculature.

*Excretory system*. Flame cells, nephridioducts, and nephridiopores not found.

*Nervous system*. Dorsal and ventral brain commissures 13 µm (9 µm in allotype) and 10 µm (7 µm in allotype) in thickness, respectively (Fig. 3).

*Vascular system*. Pair of cephalic vessels meeting above rhynchodaeum, posteriorly passing through cerebral ring (Fig. 3), extending further backward as lateral vessel on each side, situated near lateral nerve cord (Fig. 4A).

*Sensory system*. No eyes. No cerebral organs. No frontal organ.

**Figure 5.**
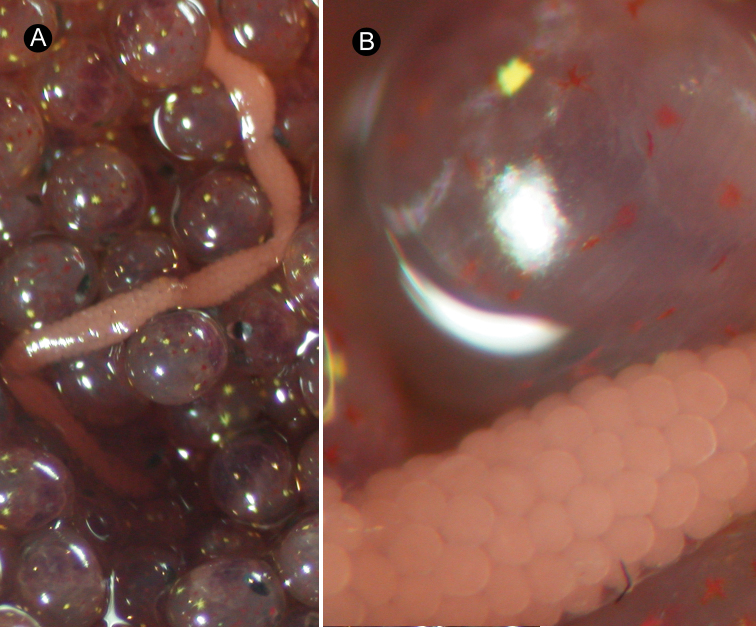
*Ovicides paralithodis* sp. n. **A** egg strand laid by holotype **B** magnification of **A.**

*Reproductive system*. Ovaries more or less regularly interspersed with intestinal lateral diverticula, arranged in row on each side of body; single oviduct from each ovary extending dorsally (Fig. 4B). Single egg string found in the same crab egg mass about 1 cm in length, containing pink eggs (Fig. 5A, B). Takakura’s duct present in male, about 40 µm in diameter (Fig. 4A).

#### Behaviour.

Fed on *Paralithodes camtschaticus* eggs *in vitro*, piercing the egg membrane with its stylet and consuming the contents of the ruptured eggs. *In vivo* feeding confirmed by frequent observations of gut contents containing crab egg yolk and eye placodes. Juvenile worms were recovered from two of 30 male and non-ovigerous female crabs collected at Juneau and Seward, Alaska. The presence of juvenile worms on hosts lacking eggs suggests that the life cycle of *Ovicides paralithodis* may be more similar to carcinonemertids such as *Carcinonemertes errans* Wickham, 1978 where worms can transfer from males to females, and from premoult to postmoult cuticles of non-ovigerous crabs (Wickham et al. 1984, Kuris 1993) than to *Carcinonemertes regicides* of the red king crab for which transmission only occurs among brooding female crabs (Kuris et al. 1991). A life cycle involving non-ovigerous hosts may be common among *Ovicides* spp. since Shields and Segonzac (2007) described the other known species of *Ovicides* from non-ovigerous crabs.

#### Ecology.

The proportion of infested crabs exceeded 50 percent at 13 localities in Alaska, reaching 100 percent at five localities. At six localities the intensity of infestations exceeded 1,000 worms per pleopod (red king crabs have six egg-bearing pleopods), with the highest reported intensity at Terror Bay, Kodiak Island, >24,000 worms per pleopod (Kuris et al. 1991) (voucher specimens are deposited in the Santa Barbara Museum of Natural History, CA, USA). At most locations sampled in Alaska it co-occurred with *Carcinonemertes regicides*, but it was usually less abundant than *Carcinonemertes regicides*. It was the only symbiotic egg predator nemertean present on red king crabs along the Alaska Peninsula and it was rare at Cook Inlet where *Carcinonemertes regicides* caused up to 95% brood mortality.

#### Etymology.

The specific name, *paralithodis*, is a noun in the genitive case, derived from the generic name of the host crustacean, *Paralithodes camtschaticus*.

#### Distribution.

In addition to the type locality, the Sea of Okhotsk, off Abashiri, Hokkaido, Japan, *Ovicides paralithodis* has been reported from Adak, Dutch Harbor, Morshovoi Bay, Pavlof Bay, Kodiak Island, Resurrection Bay, Seward, Cook Inlet and Southeastern Alaska (Barlow Cove, Deadman’s Reach, Gambier Cove, and Pybus Cove, Juneau) by Kuris et al. (1991) as Form 4. The distribution of *Ovicides paralithodis*, may generally overlap the native range of its host, *Paralithodes camtschaticus* although it is apparently absent over some large areas such as Bristol Bay and Norton Sound, Alaska. The red king crab was intentionally introduced into the Barents Sea, northern Europe, from the northern Pacific in 1961–1969 (Orlov and Ivanov 1978), and its distribution now extends westward beyond the Kola Peninsula to the Norwegian coast (Falk-Petersen et al. 2011) and north to the Svalbard archipelago (Kirby 2003). Surveys of the introduced Atlantic population of *Paralithodes camtschaticus* for epifauna and parasites have not recovered any symbiotic egg predator nemerteans (Dvoretsky and Dvoretsky 2010, Falk-Petersen et al. 2011). Apparently the introduced crabs were not infested with these important natural enemies. This lack of infectious natural enemies may contribute to their rapid population growth and geographic expansion in the northeastern Atlantic Ocean (Torchin et al. 2003, Falk-Petersen et al. 2011).

#### Taxonomic remarks.

Of the four currently recognised congeners in *Ovicides*, *Ovicides paralithodis* is distinguished from *Ovicides julieae* and *Ovicides davidi* by the absence of eyes. *Ovicides jasoni* and *Ovicides jonesi* are eye-less as is the new species. *Ovicides jasoni* can be distinguished from *Ovicides paralithodis* in having densely arranged submuscular glands (Shields and Segonzac 2007, fig. 3E). *Ovicides jonesi* differs from the new species in that it lacks vacuolated glandular lobes in the precerebral region (Shields and Segonzac 2007, fig. 6B–D). The new species differs from *Ovicides julieae* also in that the lateral vessels fuse above the oesophagus (seemingly postcerebrally, cf. Shields 2001, fig. 1) in the latter, while *Ovicides paralithodis* has a pair of precerebral cephalic vessels, which meet above the rhynchodaeum, posteriorly passing through the cerebral ring. The markedly different habitats of the hosts (hydrothermal vents and tropical coral reef for the previously described species of *Ovicides* versus boreal continental shelf waters for *Ovicides paralithodis*) and the very different types of hosts (brachyuran crabs versus an anomuran) add to the distinctive nature of the present species. The dorsal position of the ovarian pore in *Ovicides paralithodis* seems to be unique in Carcinonemertidae.

*Ovicides paralithodis* has only been confirmed from *Paralithodes camtschaticus*. However, a similar eyeless form with accessory stylet pouches is common on tanner crab, *Chionoecetes bairdi* Rathbun, 1924 and has also been found on the Dungeness crab, *Cancer magister* Dana, 1852 in Alaskan waters (AMK, unpublished observations).

#### Molecular phylogeny.

**Figure 6.**
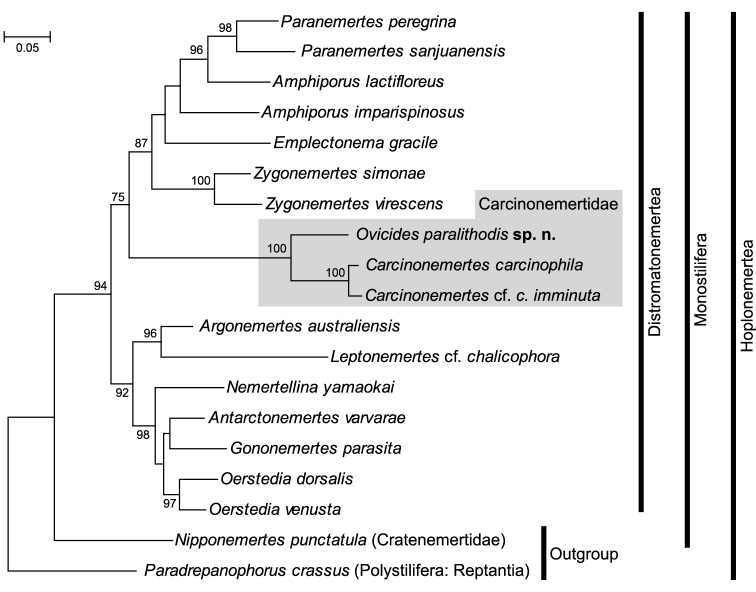
Phylogenetic tree resulting from maximum likelihood analysis of combined 28S rRNA and COI (*ln* L = –9804.30). Numbers above/below nodes indicate bootstrap support values >50%.

In the maximum-likelihood tree (*ln* L = –9804.30) (Fig. 6), *Ovicides paralithodis* appeared as a sister taxon to the clade comprised of *Carcinonemertes carcinophila* (Kölliker, 1845) of Andrade et al. (2012) and *Carcinonemertes* cf. *carcinophila imminuta* Humes, 1942 of Thollesson and Norenburg (2003). The clade comprised of these three species (family Carcinonemertidae) was supported by 100% bootstrap value.

## Discussion

One may infer from the present tree topology that the acquisition of Takakura’s duct and the loss of cerebral organs occurred in the common ancestor of the family, prior to the loss of accessory stylet pouches or stylets, which happened only in the lineage leading to *Carcinonemertes*, but not in *Ovicides*. We conclude so because 1) Takakura’s duct is possessed by all carcinonemertids, and is otherwise unique in the phylum, 2) with a few exceptions, monostiliferans generally possess cerebral organs, and 3) accessory stylets and their pouches are widespread features among Hoplonemertea, including *Ovicides*, but are absent in *Carcinonemertes*. An implication of this character-evolution scenario is that the genus *Ovicides*, currently diagnosed as a nemertean egg predator having accessory stylets (a plesiomorphy for Carcinonemertidae), may not be monophyletic.

This study supports monophyly of Carcinonemertidae, in agreement with the views of Wickham and Kuris (1988) and Shields et al. (1989). In addition to the characters commonly found among carcinonemertids such as the absence of cerebral organs or the presence of Takakura’s duct, Humes’ (1942) original diagnosis of the family also included 1) one central stylet, 2) no accessory stylet pouches or stylets, 3) anterior proboscis chamber small and non-glandular, and 4) excretory apparatus absent. Wickham and Kuris (1988) pointed out a necessity to loosen the familial diagnosis, because their Form 4, herein described as *Ovicides paralithodis*, did possess accessory stylet pouches (and accessory stylets) and Takakura’s duct. Upon the discoveries of the excretory system in *Carcinonemertes regicides* and *Carcinonemertes epialti* Coe, 1902, as well as the large anterior proboscis chamber in *Carcinonemertes regicides*, Shields et al. (1989) emended the diagnosis by removing the above-mentioned four characters about stylets, anterior proboscis chamber, and excretory system. Shields et al. (1989) regarded the following five characters as diagnostic for the family: 1) symbiotic relationship with a decapod crustacean, 2) short proboscis, 3) absence of cerebral organs, 4) presence of Takakura’s duct, and 5) a “rhabdocoel-like” [sic] hoplonemertean larva [i.e., planuliform larva]. But for the last character, which is not ascertained in *Ovicides*, all of these apply to *Ovicides paralithodis*. Presence of Takakura’s duct, however, is not confirmed in any other congeners, because no adults are known for *Ovicides davidi*, *Ovicides jasoni*, and *Ovicides jonesi* (Shields and Segonzac 2007); as to *Ovicides julieae*, which is a simultaneous hermaphrodite, Shields (2001: 305) stated that “Takakura’s duct may be present but not observed”.

The sister-taxon relationship of Carcinonemertidae among Monostilifera remains uncertain, although the search for it would have a fundamental significance in divergence-time estimates within the phylum. So far, all the carcinonemertids are symbiotic egg predators of Achelata, Anomura, and Brachyura (Jensen and Sadeghian 2005), suggesting that the ancestors of Carcinonemertidae acquired their egg-predatory life style after the host reptantic decapods split from other pleocyemates (i.e., Caridea and Stenopodidea, after Bracken et al. 2009). Fossil records indicate that a radiation of decapods occurred in Triassic–Jurassic (Schram and Dixon 2004). Therefore, carcinonemertids may also have radiated in this period at the earliest.

The position of Carcinonemertidae is likely to be susceptible to long-branch attraction. *Carcinonemertes* cf. *carcinophila imminuta* appeared as sister to all the rest of Distromatonemertea included in the analysis by Thollesson and Norenburg (2003). On the other hand, Andrade et al. (2012) showed the phylogenetic position of *Carcinonemertes carcinophila* was method-sensitive, being either the sister to Distromatonemertea (in direct optimization method) or nested among Distromatonemertea (in maximum likelihood and Bayesian analysis), with low nodal support values in both cases. In the present analysis, Carcinonemertidae was nested among Distromatonemertea, appearing to be more closely related to *Amphiporus* than to *Oerstedia* (Fig. 6).

## Supplementary Material

XML Treatment for Ovicides paralithodis
